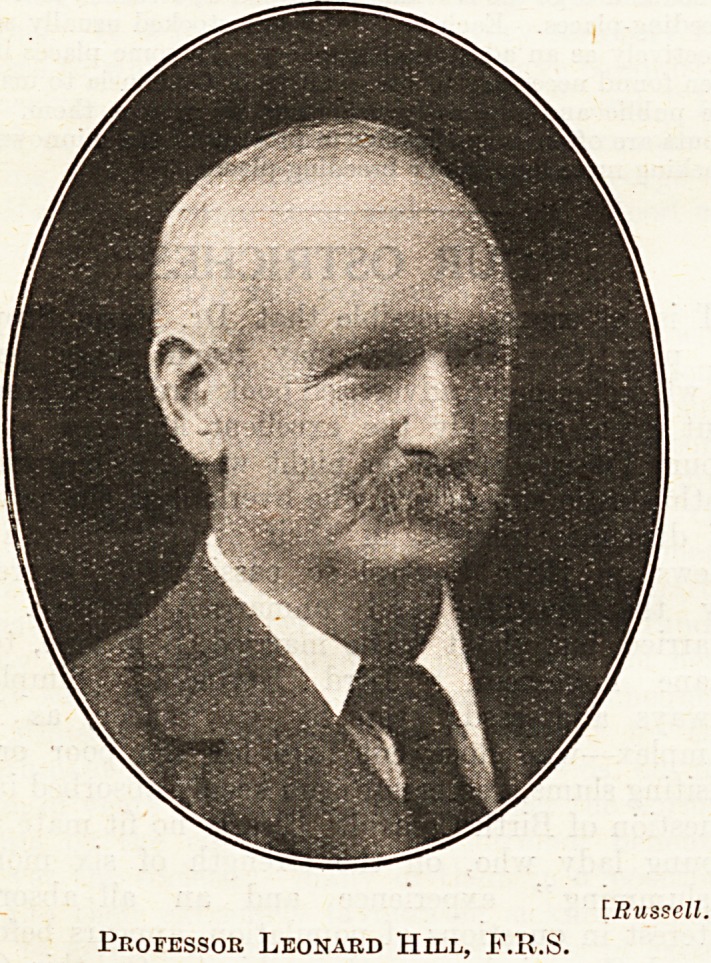# Clothing and Health

**Published:** 1923-12

**Authors:** 


### CLOTHING IN RELATION TO HEALTH.

### LECTURE BY PROFESSOR LEONARD HILL.

PROFESSOR LEONARD HILL, M.B., F.R.S., has delivered an interesting and important address on "Clothing and its Relation to Health" at Sheffield in connection with the winter's course on "Health Education" under the auspices of the Education Department, the Health Committee, the Insurance Committee, the Joint Hospitals Council and the Association of Hospital Contributors. The following is a summary of the outstanding features of the lecture.

### The Clothing of Primitive Man.

Man, he said, became evolved probably hundreds of thousands of years ago in tropical climates, in many respects differing, but in respect of a coloured

[Russell.

Professor Leonard Hill, F.R.S.

skin and hairiness probably ape-like. Becoming erect and able to use his hands for offence, and his ingenuity developing together with the fashioning of weapons, he adorned himself with the furry skin of animals, wove feathers and leaves together with threads stripped from the stems of vegetables, and finally wove wool, silk, cotton and linen fibres into clothing materials; the evidence of ancient tombs shows such weaving goes back for many thousands of years. With the aid of clothes man was able to spread into cold climates and people all parts of the earth. Clothes became the means of adornment and sexual attraction, and the indication of rank in the tribe.

### Coming Back to Greek Ideals.

How many people, now important and full of self-esteem, when stripped of their clothes and made to sit on a form would become in our sight insignificant and even contemptible? The commissionaire might be picked out as the most reputable, in place of his master, a profiteer in cheeses or chemises, or Lord Tomnoddy, the last of an aristocratic line. The Society beauty with the elegant silk stockings and tight, high heeled shoes might show ugly naked feet, her toes twisted and deformed with bunions and corns by the high heels she has always worn; and her face, robbed of the protective shadow of her hat and set in the full blaze of light, might show the rouge and powder used to disguise the complexion spoilt by neglect of air and exercise, by late hours spent in cinemas, dancing halls and bridge parties, and by an ill-chosen diet. The old Greek custom of exercises and games played in the nude state maintained health and vigour and a proper sexual selection. Girls should not be chosen for painted faces and pretty clothes. Sports and bathing costumes showing more of the figure are bringing us back to Greek ideals.

### Furs, Feathers and Clothes.

The hair and skin with its subcutaneous fat form the natural garment, and still remains so in many savage races. It is noteworthy that the inhabitants of Terra del Fuego, in South America, just north of Cape Horn, sustain naked a climate as inclement as ours. The skin secretes oil, and stinking fish oil is used by the Fuegians to amplify the natural supply in order to keep out the wet. Fur, feathers and clothes keep us warm by entangling and immobilising air, which is a very bad conductor of heat. The air caught within the meshes becomes heated by the body heat and surrounds us with a warm climate. Thus in bed, while the body temperature is 98.5° F., that of the air underneath the clothes is about 90° F.—as warm as the Tropics. The heat conductivity of water is forty times as great as that of immobilised air, that of wool fibres free from air some six times, that of linen or cotton, also free from air, thirty times. Cold water therefore feels much colder than cold air, a cotton or linen garment colder than a woollen one when first put on. The nature of the fibre, however, makes very little difference in the end, because our clothing materials are woven so as to entangle and immobilise air, and it is this air and not the fibre which keeps us warm. There is no foundation for the claims that one kind of fibre is more healthy to wear than another. Wool-fibres are covered with imbrications which—seen under the microscope—enables one easily to identify pure cotton and pure wool and mixtures of cotton and wool; so, too, linen and silk. It is the imbrications on the wool fibre which tickle the skin and may make a child, unused to them, itch all over almost unbearably when first put into woollies. The wool fibres are much more elastic than cotton or linen ones, and when wet do not cling closely to the skin but keep air entangled between them. Thus wet wool is warmer than wet cotton or linen, because more air is entangled in it. So long as the clothes are dry, both cotton and linen are just as warm as wool if of equal texture and thickness, but if wetting has to be faced wear wool, and coarse wool.

### How to Keep Warm.

In spite of their being wet, clothes can conserve a great deal of body heat, particularly if they are thick or enclosed within a waterproof. An instrument designed by me, the Kata-thermometer, enables one to measure the rate of a surface cooling at body temperature. It is an excellent measurer of ventilation, most sensitive, as we are, to the cooling effect of wind, and can be used to secure comfortable and healthy conditions. With its bulb in water it cools fourteen times more quickly than in air of the same temperature. When clothed in a thick, wet wool glove it cools much more slowly on exposure to a wind than when clothed with thin, wet muslin. If a thin rubber coat is put outside the wet wool it then cools very slowly indeed. Similarly, I have shown that a man loses much less heat when he is protected by thick, wet clothes than when naked. Far more so when he also has an outer waterproof cover on. The permeability of the clothes by wind is a very important matter, and the Kata-thermometer shows that this is so. We want our clothes to be permeable and well ventilated under ordinary conditions in order to remove the body-heat and moisture. Loosely woven materials and free openings at the neck, wrists, ankles or knees allow free ventilation. A great deal of nonsense is talked and believed generally about the danger of damp clothes and draughts. We want to live at a lively rate and be impelled to keep ourselves warm by exercise and have a vigorous appetite and well-toned-up muscles. It is only the poor who cannot afford to pay for food, who must huddle up in close tenements. Cold is a friend to the well-fed and strong, an enemy to the semi-starved and failing.

### Absorption of Light.

Another matter of great importance is the absorption of light by the clothes. My colleagues and I have just discovered that ultra-violet rays acting on the skin of a rabbit notably increase the bactericidal power of its blood. Clothes absorb light rays and convert these into heat. Black and dark clothes absorb most, while white and light colours reflect a good deal. We wear dark clothes in winter to catch warmth. In the Tropics white clothes are worn to reflect the sunlight. These must be very loose and thin to allow of free ventilation. If they are cellular some burning of the skin takes place through the meshes, and one has gradually to expose oneself to get brown. The pigment once formed in the skin stops further sun-burning by absorbing the light rays and turning these into heat, which excites sweating. The heat is then lost by evaporation of sweat.

### The Healthy Life.

The changeable climate of England has made us into a hardy, colonising race, able to stand up against Arctic and Tropic conditions. The last are far the hardest to bear by the white man. The tendency has been in recent years to produce too much tropic conditions round our bodies by over-clothing and artificial heating of houses. By such means we lower the virility of the race. Schools conducted in open air in all possible weathers and games conducted in scanty clothing, and avoidance of coddling, make a hardy, virile race. Let us see to keeping up this. We have got rid of tight lacing to a large extent, and brought girls up to games and athletic exercises, and thus, we believe, abolished to a very large extent the greensickness or anæmia to which they were so subject. Women have the advantage over men in open necks. It would be a great boon to man if the Prince of Wales would set us the fashion of the low-cut open collar, such as you see by portraits were worn at the time when Shelley wrote his poetry. Light clothing and open neck, a wide shoe with low heel and flat sole, plenty of active outdoor exercise and exposure to the sun and open air, plain, good natural food, three meals a day and nothing between-whiles, open windows in the bedroom, or, better, verandahs for sleeping on—these make healthy youngsters.

## Figures and Tables

**Figure f1:**